# Perioperative Blood Management

**DOI:** 10.3390/jcm14113847

**Published:** 2025-05-30

**Authors:** Shruti Parikh, Taylor Bentz, Samuel Crowley, Seth Greenspan, Ana Costa, Sergio Bergese

**Affiliations:** Department of Anesthesiology, Renaissance School of Medicine, Stony Brook University, Stony Brook, NY 11794, USA; shruti.parikh@stonybrookmedicine.edu (S.P.); taylor.bentz@stonybrookmedicine.edu (T.B.); samuel.crowley@stonybrookmedicine.edu (S.C.); seth.greenspan@stonybrookmedicine.edu (S.G.); ana.costa@stonybrookmedicine.edu (A.C.)

**Keywords:** perioperative guidelines, preoperative evaluation, preoperative optimization, surgical optimization, enhanced recovery, perioperative outcomes, blood management, blood products, massive transfusion protocol, TRALI, TACO

## Abstract

Perioperative blood management strategies include evidence-based guidelines to efficiently manage blood products and transfusions while minimizing blood loss and improving patient outcomes. Perioperative Medicine has made evident that anemia is often under-recognized and not appropriately addressed prior to surgery. Early recognition and correction of anemia is imperative for better surgical optimization, fewer transfusions perioperatively, and improved outcomes. Patient blood management utilize evidence-based guidelines for the establishment of a framework to promote treatment of the causes of anemia, reduce blood loss and coagulopathy as well as to improve patient safety and outcomes by efficiently managing blood products, decrease complications associated with blood transfusions and reduce overall costs. Both liberal and restrictive strategies for blood transfusions established thresholds for hemoglobin: restrictive transfusion threshold of hemoglobin 7–8 g/dL in stable patients, and a higher transfusion threshold of hemoglobin > 8 g/dL may be considered in patients with cardiac disease. Intraoperatively, tests such as viscoelastic testing, including rotational thromboelastometry and thrombelastography, offer real-time analysis of a patient’s clotting ability, allowing for targeted transfusions of fresh frozen plasma, platelets, cryoprecipitate or antifibrinolytic drugs. Complications associated with blood transfusions include allergic reactions, delayed hemolytic reactions, transfusion related acute lung injury, transfusion-associated circulatory overload, and the transmission of infectious diseases such as Hepatitis B, Hepatitis C, and Human-immunodeficiency virus. This review will discuss the management of blood products for surgical patients in the entire perioperative setting, with specific considerations for the peri-, intra- and post-operative stages.

## 1. Introduction

An adequate blood volume is essential for the preoperative optimization of a surgical patient in order to minimize intraoperative and postoperative complications. An increasingly aging population and expansion of ambulatory surgical centers have contributed to an increase in the number of surgeries performed yearly. The field of Perioperative Medicine has made evident that anemia is often under-recognized and not appropriately addressed prior to surgery. Preoperative anemia has been shown to affect surgical outcomes including increased length of hospital stay, postoperative complications, development of myocardial ischemia, infections, and overall increased surgical morbidity and mortality [1,2]. Ideally, anemia should be identified and treated as early as possible prior to surgery with identified nutritional deficiencies being treated at least 6–8 weeks prior to surgery [3]. The transfusion of red blood cell (RBC) is a common and costly treatment, with approximately 118 million units of blood being collected worldwide yearly [4,5]. Medical treatments, including blood transfusions, carry risks (Table 1).

The concept of blood health focuses on the prevention, diagnosis, and timely treatment of the causes of anemia and coagulopathies and the critical need to minimize blood transfusions, thus contributing to improved patient safety and outcomes. Patient Blood Management (PBM) programs utilize these principles of blood health to create a framework that involves a multidisciplinary approach involving public health agencies, healthcare professionals, and patients and their families to promote the prevention and treatment of anemia and coagulopathies and preserving a patient’s own blood, moving away from a reliance solely on blood transfusions [6]. The World Health Organization (WHO)’s guidance on the implementation of a PBM promotes a systematic, evidence-based, patient-centered approach to managing and preserving a patient’s own blood, which involves erythropoiesis optimization, minimizing blood loss, and maximizing one’s tolerance to anemia [7]. WHO’s PBM guidance, published in 2024, aims to promote optimal blood health worldwide, decrease costs associated with blood transfusions, and decrease healthcare inequities by reducing the global disease burden of anemia, blood loss and bleeding disorders. PBM programs have been shown to improve patient outcomes The PBM implemented by the Western Australia Department of Health resulted in a reduction in blood transfusions, hospital mortality, infection rates, myocardial infarction, stroke length of hospital stay in a 6-year period with more than 600,000 patients admitted to four major tertiary care hospitals [8]. PBM programs are critical for the improvement of patient outcomes and safety since blood product transfusions have been associated with adverse outcomes, including major morbidity and mortality [9].

A liberal approach to transfusions, with the goal to maintain hemoglobin (Hgb) levels 8–10 g/dL or higher, was used in the past [10]. A restrictive approach to blood transfusions, Hgb level (typically 7 g/dL), is considered safer and more effective in non-cardiac patients that are hemodynamically stable [11]. The American Association of Blood Banks (AABB) advocates for transfusions to be reserved for patients meeting the restrictive transfusion threshold of Hb 7–8 g/dL in stable individuals [11]. A higher transfusion threshold of Hgb > 8 g/dL may be considered in patients with cardiac disease [12]. Thus, the healthcare professional must carefully weigh the risks and benefits of a blood transfusion. Early recognition and correction of anemia is imperative for better surgical optimization, fewer transfusions perioperatively, and improved outcomes [2]. Elective surgeries for benign conditions should be delayed until anemia is appropriately treated when feasible [3]. Recent studies have suggested a possible increased bleeding risk in blood type O individuals due to lower levels of von Willebrand factor (vWF) [13]. However, further research is needed to confirm any bleeding complications in patients with type O blood. Efforts to improve transfusion practices have focused on minimizing unnecessary type and screen or crossmatch orders, aiming to reduce blood product wastage, conserve resources, lower costs, and expedite the availability of compatible blood products.

Transfusion of RBCs is an independent risk factor for morbidity and mortality, with the amount of transfused RBCs possibly posing a dose-dependent risk factor for mortality [14,15]. Thus, methods such as autologous blood transfusion, acute normovolemic hemodilution (ANH), erythropoietin (EPO) administration, and cell salvage are utilized to mitigate the risk of perioperative blood loss and decrease the need for allogenic blood transfusion. Given equivocal outcomes and the costly and limited availability of allogenic blood, practice guidelines by both the AABB and the American Society of Anesthesiologists (ASA) advocate for restrictive transfusion strategies regarding perioperative blood management [16,17]. However, various studies have been unable to determine whether a significant difference in mortality at 30 days exists between a restrictive and a liberal transfusion strategy [18].

Excessive surgical blood loss can lead to the development of a coagulopathic state, making surgical hemostasis difficult and contributing to worsening bleeding. The preoperative assessment must specifically ask the patient about any inherited bleeding disorders such as von Willebrand disease or hemophilia as well as medication history to identify drug-acquired hypocoagulopathy. The management of drug-acquired coagulopathy in the perioperative period is complex and a multi-disciplinary team is often needed to assess whether the medication can be held and for how long in the setting of surgical bleeding risk, urgency of surgery, the class of agent, and its indications. Reversal agents that restore normal coagulopathy must be considered in emergency surgeries where major bleeding is anticipated [19]. In addition to maximizing the patient’s overall health and identifying any coagulopathy preoperatively, anti-fibrinolytic medications can be utilized during surgery to help prevent surgical blood loss by inhibiting the activation of plasmin or other proteases that breakdown fibrin clots. As the CRASH-2 trial demonstrated, tranexamic acid (TXA) given to trauma patients at risk for major bleeding within 8 h of injury reduces mortality and death from bleeding [20]. Studies have shown that aprotinin, TXA, and epsilon aminocaproic acid (EACA) for elective surgery lead to a reduction in the risk of RBC transfusion [21].

Furthermore, the appropriate allogenic transfusion of plasma, platelets, and fibrinogen is essential in the management of coagulopathy in the setting of intraoperative hemorrhage. Tests such as the viscoelastic testing, including rotational thromboelastometry (ROTEM) and thrombelastography (TEG), offer a real time analysis of a patient’s clotting ability by measuring the strength, speed, and quality of clot formation. Abnormal values in subcomponents of this assay can guide physicians to provide targeted transfusions of fresh frozen plasma, platelets, cryoprecipitate, or antifibrinolytic drugs. These tests are critical in the assessment of a dynamic coagulopathy and the need for targeted transfusions. Clinical practice guidelines from trauma surgeons recommend the use of ROTEM/TEG-guided transfusions for hemorrhaging trauma patients at risk of coagulopathy [22]. In the setting of massive blood loss, a rapid infuser may be necessary to expeditiously transfuse blood products.

Any transfusion of blood products can lead to complications whether minimal or massive amounts of products are transfused. The inadvertent transfusion of ABO incompatible blood is a rare but potentially lethal outcome. Delayed hemolytic reactions may also occur when antibodies are exposed to Rh and non-ABO antigens [23]. Furthermore, transfusion related acute lung injury (TRALI) and transfusion-associated circulatory overload (TACO) are potential adverse pulmonary complications following blood transfusions. While TRALI is one of the most common causes of transfusion-related morbidity and mortality, TACO remains the most common pulmonary complication following a transfusion, with an overall incidence of 1% [24,25,26]. Treatments of these complications are mostly supportive.

Post transfusion Hb and Hb values at time of discharge are metrics used to evaluate transfusion outcomes. The implementation of an institution-wide policy to guide physicians in restrictive and liberal transfusion strategies has been shown to lead to decreased complications in the setting of more restrictive transfusion strategies. Patient blood management includes evidence-based guidelines to efficiently manage blood products and transfusions, minimize blood loss, and ultimately improve patient outcomes [27]. This review utilized PubMed Central, Scopus, and Web of Science for its literature review, including publications describing historical and modern blood management practices, and will discuss the management of blood products for surgical patients in the entire perioperative setting, with specific considerations for the peri-, intra- and post-operative stages.

**Table 1:** Complications of blood transfusion.

Adverse Reaction	Risk
Febrile Reaction	1:161
Allergic Reaction	1:345
Transfusion-associated Circulatory Overload (TACO)	1:125
Transfusion-associated Acute Lung Injury (TRALI)	1:1250
Anaphylactic Reactions	1:5000
Infections:	
Hepatitis B Virus	1:1,100,000
Hepatitis C Virus	1:1,200,000
Human Immunodeficiency Virus	1:1,600,000

Risk is approximate per RBC transfusion [28,29].

## 2. Preoperative Anemia Screening

Anemia is typically defined as a Hb concentration < 13 g/dL in men, <12 g/dL in non-pregnant women and <11 g/dL for pregnant women [2,3,30]. It is a common finding in preoperative surgical patients with an estimated prevalence of 30–40% [2,30]. However, the WHO has defined anemia as a Hb < 13 g/dL regardless of sex in the setting of high-blood-loss surgeries [31]. The prevalence of anemia varies depending on age, sex, nutritional status, comorbid conditions, the underlying reason for surgery, geographic area, and other contributing factors. Post-operatively, anemia becomes even more prevalent, affecting up to 90% of patients [1]. Substantial evidence links preoperative anemia to adverse surgical outcomes including increased length of hospital stays, post-operative complications, development of myocardial ischemia, infections, and overall surgical morbidity and mortality [1,2]. Moreover, preoperative anemia is a strong independent predictor of perioperative blood transfusion, which itself carries inherent risks [30]. Despite its clinical significance, anemia is often under-recognized and inadequately addressed during surgical planning.

The Enhanced Recovery After Surgery Society recommends routine screening and treatment of preoperative anemia [32]. This is especially important in surgical procedures where there is expected to be moderate to high amounts of blood loss. While the causes of anemia are varied, about one-third are due to nutritional deficiencies—primarily iron, but also folate and vitamin B12 [32]. Anemia can also be directly related to the reason for undergoing surgery, such as gastrointestinal bleeding, or a consequence of chronic disease [2]. Collecting a detailed patient history is crucial and should include any history or symptoms of anemia, history of bleeding or coagulopathies, transfusion history, family history of bleeding, and careful review of any antithrombotic medications used [1,12]. Initial laboratory evaluation should include a complete blood count, iron studies (iron concentration, total iron-binding capacity, transferrin saturation, ferritin), reticulocyte index, and levels of folate and B12 [1,2]. Additional labs may include markers of inflammation such as C-reactive protein and renal function if anemia of chronic disease or chronic kidney disease are suspected [3]. Coagulation studies—activated partial thromboplastin time (PTT) and prothrombin time (PT)/international normalized ratio (INR)—can help to predict bleeding risk during the procedure [12].

Ideally, anemia should be identified and treated as early as possible prior to surgery. The Network of Advanced Transfusion Alternatives recommends screening at least 4 weeks before surgery [12]. If nutritional deficiency is identified, supplementation (oral or intravenous) should be started as far as 6–8 weeks prior to surgery [3]. Blood transfusions should be reserved for patients meeting the restrictive transfusion threshold of 7–8 g/dL in stable individuals as recommended by the AABB [11]. In patients with cardiovascular disease or who require an orthopedic procedure, blood transfusions should be administered with a goal Hb of 8 g/dL or higher [17]. Early recognition and correction of anemia leads to better surgical optimization, fewer transfusions, and improved outcomes [2]. When feasible, elective surgeries for benign conditions should be delayed until anemia is appropriately managed [3].

## 3. Preoperative Risk Stratification

While preoperative anemia is a significant and modifiable risk factor for poor surgical outcomes, several other variables should be considered when risk-stratifying patients. Bleeding risk is influenced by the type and duration of surgery, prior operations at the same site, the surgeon’s skill, and the degree of tissue trauma involved [12]. As surgical techniques have continued to improve with less invasive laparoscopic/robotic techniques and improved hemostatic agents, there has been a more judicious use of blood products. Efforts to improve transfusion practices have focused on minimizing unnecessary type and screen or crossmatch orders, aiming to reduce blood product wastage, conserve resources, lower costs, and expedite the availability of compatible blood products [12,33]. The Maximum Surgical Blood Ordering Schedule was developed based on historical data of procedure-specific transfusion requirements and estimated blood loss to help guide preoperative laboratory ordering [33,34]. Depending on the procedure, it outlines whether no pre-operative blood work is needed, or if a type and screen or type and crossmatch is required, including the recommended number of units of RBC (typically 2 or 4). One limitation of this approach is that it may not account for individual patient factors such as pre-existing anemia [35]. In such cases, even if the procedure has a historically low transfusion rate, it may still be appropriate to order a type and screen or crossmatch, depending on an educated assessment of the patient’s comorbidities and likelihood of bleeding perioperatively.

The preoperative evaluation should include a thorough transfusion history, including any prior adverse reactions and known alloantibodies that may have developed from previous transfusions or pregnancies. If either circumstance is present, this may delay the process of obtaining adequate products that may need to be manipulated or manually serologically crossmatched, which could delay surgical start time or put the patient at risk if an emergency [12]. In emergencies, type O blood is often used as a universal donor. However, blood products should be ABO- and Rh-compatible and fully serologically crossmatched, whenever possible [33]. A type and screen test, which takes approximately one hour, determines the patient’s ABO and RhD type and screens for clinically significant antibodies. If antibodies are detected, a crossmatch is required to ensure donor–recipient compatibility, which may take an additional hour or more [12]. While same-day admission testing is common practice and works well for most, about 2% of patients are found to have unexpected alloantibodies [36]. Depending on the specific antibody and local blood bank inventory, locating antigen-negative red blood cells may take several hours—or longer in rare cases [12]. To mitigate these delays, a pre-admission type and screen performed 6–28 days before surgery is recommended when appropriate [36].

Although still under investigation, emerging studies have suggested a possible association between blood type O and increased bleeding risk. Type O individuals—who comprise the most common blood group—have 25–35% lower levels of vWF compared to other blood types, which is critical for coagulation [13]. Recent studies have investigated this association and have reported a link to an increased risk of moderate perioperative blood loss (defined as a Hb drop > 3 g/dL or hematocrit drop > 9%), although no significant increase in severe bleeding or transfusion requirements was found [37]. A 2022 study associated type O patients with increased hyperfibrinolysis and massive transfusion required post-injury [13]. Type O individuals have also been associated with increased mucosal bleeding, slightly elevated risk of postpartum hemorrhage, epistaxis, and secondary bleeding after tonsillectomy [37]. However, other studies have found no association between bleeding complications, so further investigation is warranted, especially considering type O blood is widely used in initial resuscitations when the patient’s blood type is unknown.

Other populations warranting special risk stratification include women, who are at greater risk of postoperative anemia due to lower baseline circulating blood volume, so the same amount of blood loss can have a larger effect comparatively [1,38]. Patients with sickle cell disease may benefit from higher Hb transfusion thresholds (9–10 g/dL) to reduce post-operative complications [39,40]. In certain populations, studies suggest that preoperative transfusions in patients with sickle cell disease may decrease the risk of postoperative acute chest syndrome [40]. Furthermore, special consideration must be given to Jehovah’s Witnesses, who may require extended preoperative optimization due to their refusal of blood products on religious grounds.

## 4. Perioperative Risk Mitigation

While anemia is a risk factor for morbidity and mortality in the perioperative setting, transfusion of RBCs has also been shown as an independent risk factor for morbidity and mortality [14]. As such, various methods have been investigated to mitigate the risk of perioperative blood loss and decrease the need for allogenic blood transfusion. Such methods include autologous blood transfusion, ANH, erythropoietin administration, and cell salvage. Preoperative autologous blood donation (PAD) is a method of surgical blood conservation that peaked in popularity in 1993 but has since declined [41]. Declining use of PAD has been attributed to several factors including decreased infectious risk of allogenic transfusion, difficult optimal timing of PAD, and poor cost effectiveness of PAD [42]. In the most recent practice guidelines for perioperative blood management from the ASA, experts agreed that PAD should only be offered if there is adequate time for erythropoietic reconstitution, which takes approximately four weeks [16].

ANH is a practice aimed at reducing intraoperative blood loss by reducing a patient’s red cell mass [43]. This is achieved with the controlled removal of whole blood from a patient and replacement of intravascular volume with intravenous crystalloid fluid. The surgery is started in the hemodiluted patient, with less loss of red cells because of an artificially lowered hematocrit [44]. Finally, the patient’s autologous blood is reinfused when surgical bleeding has abated. ANH is a technique that has been shown to decrease the risk of allogenic blood transfusion in cardiac and non-cardiac surgery [45,46,47,48]. A 2015 meta-analysis looking at 63 studies in cardiac and non-cardiac surgeries found that risk of allogenic transfusion was reduced in the ANH group compared to control by 26% (RR 0.74 95% CI 0.63–0.88) [48]. A 2020 meta-analysis looking at ANH in CABG only also found a significant reduction in need for allogenic blood transfusion [38]. These studies, however, show no significant difference in mortality, and findings are limited given significant heterogeneity. ANH is generally avoided in a few circumstances such as hemodynamic instability, presence of arrhythmia or infection, impaired cardiac function, baseline Hb < 11 g/dL, and impaired renal function with oliguria [49].

In certain circumstances, administration of EPO prior to surgery may be appropriate. Good candidates for EPO include patients with renal insufficiency, anemia of chronic disease, or who refuse blood transfusions [16]. A meta-analysis looking at the effects of perioperative EPO administration on acute kidney injury (AKI) and the need for transfusion among cardiac surgery patients found that preoperative administration of EPO was able to substantially reduce the risk of AKI and RBC transfusion, decreasing the length of hospital stay [50]. However, other studies have shown that EPO administration may increase the risk of thrombosis and mortality, thus routine use outside of specific patient populations is not recommended [51].

Finally, intraoperative blood salvage techniques can be used in operations where there is concern for blood loss > 1000 mL or if allogenic transfusion is likely. Cell salvage is also an important intervention to mitigate risk of blood loss for patients who refuse allogenic blood [52]. A 2023 meta-analysis evaluating 106 randomized trials identified several contexts in which cell salvage results in a reduction in the need for allogenic transfusion [53]. This meta-analysis concluded that there is evidence for probable reduction in the need for transfusion for cardiovascular surgery with and without cardiopulmonary bypass, and spinal surgery alone. Evidence was inconclusive for studies involving cancer, vascular surgery involving major blood vessels, hip replacement, and knee replacement. For obstetric surgery, specifically Caesarian section, there was no difference in the average amount of allogenic blood transfusions needed whether cell salvage was used or not. Overall, there was no increased risk of adverse events due to cell salvage techniques [53].

Another large meta-analysis of 75 studies found an overall reduced rate of need for allogenic RBC transfusion when cell salvage was used by a relative 38% overall, with relative risk reductions of 54% in studies examining orthopedic surgery patients and 23% for cardiac surgery patients [54]. Again, there was no significant effect on adverse events when cell salvage was used. A 2009 meta-analysis of 31 studies of patient’s undergoing cardiac surgery found that use of cell salvage techniques significantly reduced the need for transfusion of any allogenic blood product, RBC transfusion, and decreased mean volume of blood products transfused per patient, with no observed difference in mortality or post-operative adverse events between groups [55].

Thus, cell salvage techniques remain an attractive option for mitigating the risk of anemia in the perioperative setting. However, contraindications to the use of cell salvage exist. These include the presence of certain fluids that may cause toxicity, hemostasis, or cell lysis, such as clotting agents, betadine, bone cement, hypotonic fluids, etc. Relative contraindications include an active infection, active malignancy, or high risk of bacterial contamination [56,57]. The Association of Anaesthetists guidelines recommend use of cell salvage in the setting of infection or malignancy be made on a case-by-case basis with informed consent obtained pre-operatively [58].

## 5. Coagulopathy Management

Excessive surgical blood loss can lead to the development of a coagulopathic state that can contribute to worsening bleeding and a failure to achieve surgical hemostasis. In addition, any preoperative hypocoagulable states, whether drug-induced or from preexisting disorders such as hemophilia, must be considered carefully in the perioperative management of any patient. Hemorrhagic shock due to trauma, for example, causes a unique pathologic hypocoagulable state early after injury due to the synergistic effects of extensive tissue injury and excessive blood loss [19,59]. Other perioperative conditions and surgeries associated with significant coagulopathy include cardiac surgery requiring cardiopulmonary bypass, liver transplantation, postpartum hemorrhage, sepsis, and malignancy [19,59]. Key intraoperative management principles of addressing perioperative coagulopathy during hemorrhagic shock include reversing anticoagulant drugs, transfusing lost plasma proteins and platelets, performing coagulation laboratory assessment such as viscoelastic testing, and administering antifibrinolytic therapy with TXA when appropriate [60].

### 5.1. Evaluation of Preoperative Coagulopathy

As part of the assessment of all patients undergoing surgery, preoperative history taking should evaluate for any inherited bleeding disorders such as von Willebrand disease or hemophilia as well as medication history to identify drug-acquired hypocoagulopathy. The treatment of von Willebrand disease, characterized by low levels of functional vWF, is complex and varies by disease and surgery type. However, some general principles involve the use of desmopressin for patients with mild to moderate disease that are responsive, as well as measuring vWF and Factor VIII levels and function throughout the perioperative period to guide the need for vWF product utilization and dosing [61]. For patients with hemophilia A or B and inhibitors, the mainstay of treatment involves giving recombinant factor VIIa through the perioperative period as it has demonstrated hemostatic efficacy [62]. While major inherited bleeding diatheses usually present before adult, there is a low sensitivity for detecting minor bleeding disorders even when utilizing standardized assessments and laboratory testing [19]. Management of drug-acquired coagulopathy in the perioperative period is complex and decisions to hold anticoagulation should be individualized in consideration of surgical bleeding risk, urgency of surgery, the class of agent, and its indications. However, in emergency surgery where major bleeding occurs or is anticipated, such as surgical trauma or cardiac surgery, reversal agents should be strongly considered [19].

### 5.2. Anti-Fibrinolytic Drugs

Anti-fibrinolytic drugs prevent surgical blood loss by inhibiting the activation of plasmin or other proteases that breakdown fibrin clots. The CRASH-2 trial was a landmark randomized controlled trial that found a reduction in overall mortality and death from bleeding when TXA is given to trauma patients at risk of major bleeding within 8 h of injury without increased risk of thrombotic events [20]. A Cochrane systematic review including 252 randomized controlled trials evaluated the use of aprotinin, TXA, and EACA for elective surgery, and found a relative reduction in the risk of red blood cell transfusion of 34%, 39%, and 29%, respectively, when compared to placebo control [21]. However, none of these agents were associated with overall mortality differences for elective surgery, although aprotinin was associated with increased mortality when compared directly to the other agents [21]. Meta-analyses for specific surgery types have found the benefit of TXA at reducing the need for blood transfusions and 30-day mortality for hemiarthroplasty patients, and reduced morbidity for spine surgery and mastectomies [63,64,65]. A joint guideline from the Society of Thoracic Surgeons and the Society of Cardiac Anesthesthesiologists provides a class 1, level A recommendation to use TXA or EACA to reduce bleeding and the need for transfusion during cardiac surgery [66].

## 6. Viscoelastic Testing

Essential management of coagulopathy in the setting of intraoperative hemorrhage involves appropriate allogenic transfusion of plasma, platelets, and fibrinogen. The conventional laboratory tests for coagulopathy include PT or INR, PTT, fibrinogen level, and platelet count, which are reliable tests but are limited clinically by a lengthy turnaround time of typically 30–60 min [60]. These concerns have led clinicians to utilize novel real time evaluations of hemostatic function such as viscoelastic testing including ROTEM and TEG to guide resuscitation during major hemorrhage. The values of a viscoelastic signal from a whole blood sample are affected by every stage of the formation and breakdown of a clot, including thrombin production, fibrin cross-linking, platelet binding to fibrin clots, and fibrinolysis [60]. Guided algorithms based on abnormal values in the assays allow clinicians to provide targeted transfusions of fresh frozen plasma, platelets, cryoprecipitate (to replete hypofibrinogenemia), prothrombin complex concentrate or antifibrinolytic drugs/reversal agents [67,68].

Kvisselgaard et al. found in a recent meta-analysis of 31 randomized trials on bleeding surgical patients (with a majority undergoing elective cardiac surgery) that TEG or ROTEM guided algorithms reduced the number of transfusions of plasma and platelets, as well as reduced surgical reintervention and bleeding, although there was no overall mortality benefit across the pooled cohort [69]. However, a randomized controlled trial on 111 emergency trauma patients who met criteria for massive transfusion, found a reduction in mortality when clinicians utilized a transfusion protocol based on TEG rather than conventional coagulation assessments (PT, PTT, platelet count, etc.) [70]. This study and others have consequently led to clinical practice guidelines from trauma surgeons recommending the use of ROTEM/TEG guided transfusions for hemorrhaging trauma patients at risk of coagulopathy [22]. In addition to trauma, viscoelastic testing has been utilized extensively for cardiac surgery and liver transplantation due to improved morbidity, although there is less evidence for a benefit for postpartum hemorrhage [71,72].

## 7. Rapid Transfusion

In the setting of excessive blood loss, massive transfusion protocol (MTP) is often necessary. While massive transfusion refers to the administration of at least 10 units of whole blood or packed red blood cells (PRBCs) within 24 h, MTP involves rapid transfusion of all blood component products in a hemorrhaging, hemodynamically unstable patient [73,74]. MTP has been historically researched in the trauma population, but it has applications far beyond trauma including gastrointestinal, surgical and obstetric bleeding [74]. The most common cause of massive bleeding is non-trauma surgical bleeding, such as in surgeries for ruptured abdominal aortic aneurysms [74]. When rapidly administering blood components in these scenarios, the ratio in which they are administered may determine patient outcomes. The Trauma Quality Improvement Project of the American College of Surgeons recommends transfusing PRBCs and plasma in 1:1 or 1:2 ratios and to transfuse one unit of platelets for every six units of PRBCs [75]. Newer trends advocate for transfusion ratios of 1:1:1 or 1:1:2 for plasma, platelets, and PRBCs, respectively [66]. Introducing plasma platelets earlier in the transfusion process has shown to improve survival and decrease coagulopathy [75]. The Pragmatic, Randomized Optimal Platelet, and Plasma Ratios (PROPPR) clinical trial showed no significant difference in 24 h and 30-day mortality between patients transfused at 1:1:1 versus 1:1:2 [76]. Death due to exsanguination was lower in the 1:1:1 group while all-cause death was similar at 24 h [11]. In addition, a meta-analysis showed no benefit of a 1:1:1 ratio over 1:1:2 [77].

During MTP, rapid infusion of blood products is performed by specialized devices. Three commonly used devices are the Level 1^TM^ (ICU medical, San Clemente, CA, USA), Ranger^TM^ (3M, Saint Paul, MN, USA) and Belmont^®^ rapid infusion systems (Belmont Medical Technologies, Billerica, MA, USA). All devices are capable of warming fluid and blood products with a maximum infusion rate up to 500–750 mL/min [78]. The Level 1 uses automated pneumatic pressure infusers as the driving force and the Belmont uses a semiocclusive rollerhead pump [78]. In a study comparing the Level 1 and Belmont devices, only the Belmont was able to maintain physiologic temperature in infused PRBC at high flows of 500 mL/min, while both delivered PRBC infusions at physiologic temperature at lower rates of 250 mL/min [78]. When a 10 mL bolus of air was injected into each system, the entire 10 mL of air passed through the Level 1’s air filter and made it to the distal tubing going to the patient. When this was performed with the Belmont, the system was automatically stopped upon sensing air and the air was purged from the system. No air was detected in the distal tubing going to the patient [78]. When air is inadvertently infused to a patient, there is a risk of venous air embolism (VAE). When a system does not adequately remove air from the infusate, air must be removed before it reaches to the patient [79]. Comparison of the Level 1 and Ranger systems showed significantly less air was detected in the Ranger compared to the Level 1 [79]. Given the possibility of air reaching the patient with potential for VAE, an experimental device is currently under investigation to help mitigate this risk. The vascular access line air removal device (VALARD) uses centrifugal force to separate air bubbles from a crystalloid solution with a pressure gradient that forces the air to the top of the chamber where it is removed by a filter [80]. In early experiments using the VALARD with a Belmont, no air bubbles > 10 μL were detected at various combinations of air injection and flow rates when using the VALARD, while bubbles were detected when using the Belmont alone [80]. Injection of 120 mL of air at a flow rate of 500 mL/min removed the whole bolus without a pause in flow [80]. These early experiments were conducted using crystalloid only and not with blood products. No other rapid infusers were tested besides the Belmont.

Subjecting blood products to elevated pressures and flow rates raises concerns about the integrity of these products at the time they reach the patient and the physiologic consequences that may result because of it. In a small study running 16 units of PRBCs through a Level 1 infuser, clinically insignificant levels of hemolysis at 0.05% were found in post-infusion samples [81]. Hyperkalemia is another risk associated with rapid transfusion, often attributed to hemolysis of RBCs. A study assessed the risk of hyperkalemia with Belmont rapid infusion and tested various flow rates and intravenous catheter sizes [82]. They found no significant difference in pre-infusion and post-infusion potassium values of PRBCs across all tested flow rates and catheter sizes [82]. Additionally, there was no significant difference in hemolysis scores in pre-infusion and post-infusion samples [82]. Expired units of blood were included in this study. While these had a higher pre-infusion potassium level compared to fresh blood, they also showed no significant change in post-infusion potassium when run through the Belmont [82].

Since blood is refrigerated for storage, longer duration of cold storage time will lead to potassium precipitating from RBCs [83]. Furthermore, infusion of cold blood without warming can lead to hypothermia, which comes with its own harmful sequelae [83]. Hypothermia with rapid transfusion may lead to life-threatening arrythmias, citrate toxicity, delayed medication metabolism, delayed emergence, and coagulopathy [83]. In documented cases of cardiac arrest due to hyperkalemia during massive transfusion, concurrent physiologic disturbances including acidosis and hyperglycemia likely contributed to elevated potassium levels [84]. In this scenario, the presence of hypothermia or hypocalcemia from citrate toxicity would increase the risk of potassium-induced cardiotoxicity [84]. A study using cooled whole blood in a Belmont infuser showed no significant decrease in red cell count, Hb or hematocrit post-transfusion; however, there was a mean decrease in platelet count by 20% [85]. TEG analysis of the post-infusion samples showed a significant decrease in the reaction time, indicating a faster clot initiation, with a decrease in maximum amplitude and clot strength, indicating issues with platelet function, a clotting factor deficiency, or decreased clot stability [85].

## 8. Complications of Transfusion

There is potential for transfusion related complications whether minimal or massive amounts of products are transfused. A rare but potentially lethal outcome can occur with the inadvertent transfusion of ABO incompatible blood. Anti-AB antibodies spontaneously develop in all patients except those with type AB blood [86]. These antibodies will bind to incompatible RBCs and activate complement leading to acute hemolysis within 24 h of transfusion [23,86]. Patients will present with symptoms including fever, chills, hypotension, flank pain, anuria and hematuria [87]. Hemolysis leads to the release of potassium and Hb, which can ultimately lead to life threatening disseminated intravascular coagulopathy and multiorgan failure [23,86]. Delayed hemolytic reactions may also occur when antibodies are exposed to Rh and non-ABO antigens [23]. Delayed hemolysis can begin 3–30 days after transfusion due to partial activation of the complement system leading to a staged destruction of RBCs with minimal release of Hb [23]. Delayed hemolytic reactions rarely progress to medical emergencies and patients will typically present with anemia and jaundice [23]. A common cause of delayed hemolytic reactions is the development of alloantibodies from previous transfusion or pregnancy [88]. Alloimmunization occurs during the initial exposure to transfused RBCs and during subsequent transfusion the alloantibodies will react and cause delayed hemolysis [88]. There are hundreds of RBC antigens in every unit; however, a minority of patients develop alloantibodies [88]. Patients who have received multiple transfusions may develop multiple types of alloantibodies, making cross match difficult during future transfusions [88]. The process of alloimmunization is also possible with platelet transfusion, which would manifest as platelet refractoriness during future platelet transfusions [89,90]. Leukoreduction has helped decrease the risk of platelet alloimmunization [90].

Adverse pulmonary complications after blood transfusion include TRALI and TACO [81]. Both conditions begin within 6 h of transfusion and both present with respiratory distress, hypoxia, and pulmonary infiltrates on chest radiograph [25,91]. TRALI is mediated by antibodies from donor plasma that cause capillary leak resulting in lung injury with a historical incidence of 0.1% in transfused patients [91]. The rate has since lowered to 0.0008% to 0.001% [91]. TRALI is still one of the most common causes of transfusion related morbidity and mortality and remains under-diagnosed and under-reported [24]. The presence of pretransfusion inflammation plays a role in the development of TRALI known as the two-hit hypothesis [24,91,92]. With ongoing systemic inflammation, recipient neutrophils are primed for activation by the antibodies from donor plasma releasing cytokines, reactive oxygen species and proteases that disrupt the alveolar-capillary barrier resulting in pulmonary edema [24,91]. Mitigation strategies have been developed to decrease incidence and morbidity due to TRALI, and management remains as supportive care [92]. Donor selection has shifted to male-only donors due to higher antibody levels in multiparous women [92]. Since this change, the incidence of TRALI has significantly decreased in all countries that adopted this policy [92]. Management consists of supplemental oxygen, hemodynamic support with fluid and vasopressors with mechanical ventilation if necessary [25,92]. In severe cases ECMO may be required [92].

TACO remains the most common pulmonary complication after transfusion with an overall incidence of 1% which increases to 8–11% in elderly and critically ill patients [25,26]. As with TRALI, TACO will present with acute respiratory distress and pulmonary edema. However, TACO will typically include signs of cardiogenic overload such as left heart failure, elevated blood pressure and tachycardia [25]. BNP levels are more likely to be elevated with TACO than with TRALI [91]. Echocardiography is a useful tool as signs of fluid overload in TACO can be seen on imaging [91]. TACO develops when intravascular volume increases from transfusion leading to an increase in hydrostatic pulmonary capillary pressures, which leads to transudate entering the lung interstitium and alveolar space [26,91]. TACO has a two-hit model to explain its pathogenesis [25,26]. The first hit accounts for patient factors that would impair their ability to compensate for increases in intravascular volume [25,26]. These factors include extremes of age, pre-existing heart failure, need for renal replacement therapy and baseline positive fluid balance [25,26]. The second hit is blood product transfusion, which adds more volume to a system unable to accommodate [25]. Like TRALI, management for TACO is largely supportive. Treatment consists of diuretics and supplemental oxygen [25].

## 9. Outcomes and Post-Transfusion Care

Despite efforts to reduce perioperative blood loss, allogenic blood transfusions are often needed. As stated previously, allogenic RBC transfusion represents an independent risk factor for morbidity and mortality [14]. A 2014 analysis of patients undergoing elective spine surgery showed that transfusion of even one unit of PRBC or whole blood was associated with prolonged length of stay, postoperative complications, and increased 30-day return to OR [93]. These findings were independent of preoperative hematocrit level, length of surgery, and patient comorbidities, with authors concluding that increased risk was associated with transfusion, and not just a manifestation of poorer health status in transfused patients at baseline [93].

After intraoperative transfusions, assessing postoperative outcomes gives insight as to whether transfusions fell short, were adequate or potentially in excess. Post transfusion Hb and Hb at the time of discharge are metrics used to evaluate transfusion outcomes. Thus, significant investigation has been conducted to establish when allogenic transfusion is appropriate. Generally, studies have investigated a more liberal versus restrictive threshold for transfusion of blood assessing outcomes in various surgical contexts (Table 2). A 2015 review evaluated six trials assessing a liberal transfusion goal (transfuse below 10 g/dL) versus restrictive (transfuse below 8 g/dL) for hip fracture surgery patients [94]. This review found no difference in mortality seen between groups at 30 or 60 days. This review did, however, show low quality evidence for lower incidence of myocardial infarction in the liberal transfusion group. In 2018, a systemic review of patients undergoing hip or knee orthopedic surgeries with the same liberal versus restrictive transfusion goals found that a restrictive transfusion strategy is associated with increased cardiovascular events irrespective of preexisting cardiac disease, with significant findings in those undergoing hip fracture surgery, but not elective arthroplasty [95]. There was no difference in mortality or other secondary outcome measures in this review.

In 2020, a meta-analysis analyzed the question of appropriate transfusion threshold among cardiac surgery patients [96]. Ten trials and eight comparison publications were included looking at a primary outcome including mortality for the longest reported follow up; secondary outcomes included: (1) proportion of patients with new onset myocardial infarction (MI), as defined by each study; (2) proportion of patients requiring renal replacement therapy/new onset hemodialysis; (3) proportion of patients with new onset focal neurologic deficit; (4) intensive care unit (ICU) length of stay (days); (5) hospital length of stay (days); and (6) days on mechanical ventilation. Overall, there was no significant difference in mortality between the transfusion strategies and no significant adverse effects when looking at secondary outcomes [96]. Patients in restrictive strategy groups were significantly less likely to be transfused and had significantly less RBC units transfused per patient [96].

A 2020 study evaluated 19 reviews, which looked at 33 meta-analysis of mortality outcomes found that among trials with high to moderate quality evidence 75% showed no statistically significant difference in mortality between restrictive and liberal transfusion groups and 25% reported significantly lower mortality for patients assigned to a restrictive transfusion strategy [97]. Various clinical contexts including both surgical and non-surgical settings were evaluated with authors concluding that while generalizability is lacking in certain clinical contexts, it is appropriate to follow a restrictive compared to a liberal transfusion strategy as this reduces the number of patients exposed to risks of RBC transfusion and no difference in mortality exists. Another review found that a discharge Hb of 10 g/dL showed no additional benefit in outcomes compared to a Hb of 9 g/dL [10]. This suggests a post-transfusion Hb of 10 g/dL may be considered over-transfusion, where one less unit of blood could have been administered [10].

**Table 2:** Studies on restrictive versus liberal transfusion strategies.

Strategy	Transfusion Thresholds	Outcomes
Restrictive	Hgb < 7.0 g/dL Hgb < 8.0 g/dL (cardiac surgery)	Showed either no difference or decrease in mortality compared to liberal thresholds Decreased transfusion by 20–41% Decreased transfusion related complications
Liberal	Hgb < 9.0 g/dL Hgb < 8.0 g/dL	Low quality evidence for decreased risk of MI No significant improvement in morbidity or mortality

Summary of outcomes with restrictive versus liberal transfusion thresholds [10,94,95,96,97,98,99,100].

Some observational studies have shown higher morbidity and mortality when transfusion was performed at a starting Hb higher than 8 g/dL with better outcomes when the transfusion threshold was lowered from 8 to 7 g/dL [10,98]. The implementation of an institution-wide policy to lower the transfusion threshold to 7 g/dL showed a decrease in transfusions in patients with starting Hb of 8 g/dL [99]. The total percentage of patients receiving transfusion decreased and there was a decreased overall inpatient mortality rate [99]. At another institution, a similar initiative to decrease the transfusion threshold from 8 g/dL to 7 g/dL showed a 20% decrease in risk of receiving a transfusion and decreased costs due to fewer units transfused [100]. The decrease in transfusion was extrapolated to consider fewer cases of transfusion related complications and potential saved costs [100]. A meta-analysis showed a restrictive strategy using a Hb threshold of 7 g/dL showed a decrease in mortality and 40% decrease in transfusion [101]. In addition, several other adverse outcomes including acute coronary syndrome, pulmonary edema, rebleeding and infection were decreased [101]. More liberal protocols using a transfusion threshold as high as 9 g/dL showed no significant reduction in mortality or morbidity [101]. Similarly, a review of 48 trials in variety of clinical contexts, concluded that restrictive versus liberal transfusion strategy did not affect mortality at 30 days, and no significant difference in other outcomes was found [18]. Generally, restrictive strategies aimed to transfusion for Hb below 7.0 g/dL but trials involving cardiac surgery used a restrictive cutoff of 7.5 g/dL and orthopedic surgery used a cutoff of 8 g/dL. This review noted that restrictive transfusion strategy reduced the proportion of patients receiving transfusion by 41%. Given equivocal outcomes and the costly and limited availability of allogenic blood, practice guidelines by both the AABB and the ASA advocate for restrictive transfusion strategies regarding perioperative blood management [16,17].

## 10. Discussion

The management of blood products in the perioperative setting is critical for the optimization of a patient for surgery to maximize beneficial patient outcomes and minimize complications. The goals of perioperative blood management are three-fold: to optimize a patient’s blood volume, minimize blood loss, and improve the patient’s overall function in the setting of anemia before, during, and after surgery. The treatment of anemia preoperatively requires early intervention to maximize blood volume, reduce the number of transfusions and improve outcomes postoperatively. PBM programs are critical for the establishment of a framework to promote treatment of the causes of anemia, reduction in blood loss and coagulopathy as well as to improve patient outcomes by decreasing complications associated with blood transfusions and reduce overall costs. Widely accepted, the restrictive transfusion strategy, with a Hb threshold of 7–8 g/dL in hemodynamically stable patients, has been effective in minimizing unnecessary blood transfusions while maintaining adequate perfusion. However, patients with cardiac disease may benefit from the liberal transfusion strategy, allowing for a higher threshold to transfuse in the perioperative setting.

Intraoperatively, it remains critical to utilize appropriate testing methods in a dynamic fashion, as well as to have access to MTP. Viscoelastic testing methods, such as ROTEM and TEG, provide real-time analysis of a patient’s clotting ability. The results allow the physician to tailor transfusions of blood products, including fresh frozen plasma, platelets, cryoprecipitate, and antifibrinolytic drugs, to specific deficiencies experienced by the patient in real time. The targeted transfusion of blood products based on real-time analysis allows for the reduction in blood transfusions and their associated complications. Complications such as allergic reactions, delayed hemolytic reactions, TRALI, TACO, etc., highlight the importance of judicious administration of blood products. Additional considerations include the limitations for allogenic blood transfusions due to blood availability in the setting of inadequate voluntary donation and decreased as well as healthcare blood transfusion monitoring and guideline adherence.

Strategies to achieve these goals include preoperative optimization, minimizing blood loss during surgery, and implementing appropriate transfusion strategies. Perioperative blood management protocols typically emphasize the importance of early recognition and correction of anemia, judicious use of blood products, and use of strategies to reduce perioperative blood loss. These principles contribute to better patient outcomes in the perioperative setting.

## 11. Conclusions

The effective management of blood products perioperatively is imperative in the optimization of a patient for surgery and a reduction in complications. The adoption of PBM frameworks facilitate the implementation of strategies to treat the causes of anemia and reduction in coagulopathies in advance of surgical intervention, as well as the minimization of blood transfusions aimed at improving patient safety and outcomes. Early recognition and treatment of anemia, along with the implementation of a restrictive strategy for blood transfusions in stable non-cardiac patients, contribute to decreased administration of blood products as well as improved outcomes following surgery. Intraoperative strategies to reduce unnecessary transfusions and the risks associated with them include the utilization of tests in real time aimed at targeted blood product transfusions, and equipment required for massive transfusions and surgical hemostasis. Viscoelastic testing provides real-time analysis of a patient’s clotting ability and guides targeted transfusions.

Perioperative blood management strategies include evidence-based guidelines to efficiently manage blood products and transfusions while minimizing blood loss and improving patient outcomes. The adoption of these principles in the management of the perioperative patient enhances patient safety and the judicious use of blood products, a valuable resource that is often in short supply. Further research is needed to refine blood management strategies and ensure the best clinical care for surgical patients.

## Abbreviations

The following abbreviations are used in this manuscript:

AABB	American Association of Blood Banks
AKI	Acute kidney injury
ANH	Acute normovolemic hemodilution
ASA	American Society of Anesthesiologists
EACA	Epsilon aminocaproic acid
EPO	Erythropoietin
Hb	Hemoglobin
INR	International normalized ratio
MTP	Massive Transfusion Protocol
PAD	Perioperative autologous blood donation
PBM	Patient blood management
PRBC	Packed red blood cells
PT	prothrombin time
PTT	activated partial thromboplastin time
RBC	Red blood cell
ROTEM	Rotational thromboelastometry
TACO	Transfusion-associated circulatory overload
TRALI	Transfusion-related acute lung injury
TEG	Thrombelastography
TXA	Tranexamic acid
VAE	Venous air embolism
VALARD	Vascular access line air removal device
WHO	World Health Organization
vWF	von Willebrand factor
